# Association of different types of abortions with neonatal outcomes in subsequent pregnancy

**DOI:** 10.7189/jogh.14.04216

**Published:** 2024-10-18

**Authors:** Hanxiang Sun, Xiujuan Su, Jing Mao, Ruru Zhao, Qinxin Shen, Chang Zou, Yuanyuan Yang, Qiaoling Du

**Affiliations:** 1 Department of Obstetrics, Shanghai Key Laboratory of Maternal Foetal Medicine, Shanghai Institute of Maternal-Foetal Medicine and Gynaecologic Oncology, Shanghai First Maternity and Infant Hospital, School of Medicine, Tongji University, Shanghai, China; 2 Clinical Research Centre, Shanghai Key Laboratory of Maternal Foetal Medicine, Shanghai Institute of Maternal-Foetal Medicine and Gynaecologic Oncology, Shanghai First Maternity and Infant Hospital, School of Medicine, Tongji University, Shanghai, China

## Abstract

**Background:**

Abortion is an important issue that concerns all women. It holds great significance to investigate the correlation between various types of abortion histories and the neonatal outcomes of subsequent pregnancies.

**Methods:**

This retrospective cohort study included pregnant women who gave birth to singleton live-born in Shanghai First Maternity and Infant Hospital from 2016 to 2020 (n = 75 773). Women with a history of abortion, including spontaneous abortion (SAB) and induced abortion (IA), were included in the exposed group, and the remaining were included in the unexposed group. The main outcomes were birthweight and preterm birth in the subsequent pregnancy. Logistic regression models were used to estimate odds ratios (ORs) and 95% confidence intervals (95% CIs) for the association of maternal abortion history with birthweight and risk of preterm birth in subsequent pregnancy.

**Results:**

Women who have experienced SAB history had an increased risk of delivering very low birth weight (VLBW) and preterm birth children, with (OR = 1.63, 95% CI = 1.15–2.32; OR = 1.38, 95% CI = 1.07–1.79). However, women with a history of IA were at greater risk of macrosomia (OR = 1.16; 95% CI = 1.06–1.27). We also observed that the likelihood of delivering a VLBW baby was heightened by the number of SAB occurrences (OR = 0.87, 95% CI = 0.54–1.38; OR = 1.84, 95% CI = 1.01–3.36, OR = 5.71, 95% CI = 3.21–10.15).

**Conclusions:**

Our study indicates that pregnant women with a history of SAB are at an increased risk of delivering VLBW infants and experiencing preterm labour. The risk is positively associated with the number of SABs. Conversely, women with a history of IA are more likely to deliver macrosomic infants.

A global study found that there were 121 million unintended pregnancies annually between 2015–19, with 61% induced abortions [1]. Currently, many women have the option to use misoprostol and mifepristone for self-managed abortion in early pregnancy [2,3]. These are the most widely used, safe, and effective medications for self-managed abortion [4–6], with a very low probability of hospitalisation. However, the economic costs of maternal long-term morbidity, due to abortion, including retained products of conception, increased vaginal discharge and bleeding, infertility, and chronic reproductive tract infections, are significant[7–10].

Abortion can be categorised into spontaneous abortion (SAB) and induced abortion (IA). A study conducted at Boston Women’s Hospital suggested that women who experienced a history of IA were not at an elevated risk of adverse outcomes in subsequent pregnancies. In contrast, women with a history of SAB were at an elevated risk of having short gestational age and low birth weight (LBW) [11]. A study conducted in Iran also revealed that the presence of SAB history was linked to an elevated likelihood of preterm delivery, and the number of SABs was positively correlated with the incidence of preterm birth [12]. However, a study conducted in France indicated that having experienced IA increased the likelihood of preterm birth in subsequent pregnancies[13], which contradicted the conclusion of the study from Boston Women’s Hospital. Hence, there is still an inconclusive association between abortion history and adverse neonatal outcomes in subsequent pregnancies [14,15]. Additionally, there is limited data and research on this topic in Asian countries.

In this study, we aimed to explore the relationship between different types of abortion history and adverse neonatal outcomes in subsequent pregnancies. Furthermore, we sought to investigate whether the likelihood of adverse outcomes in newborns would increase as a result of an increasing number of previous abortions of various types.

## METHODS

### Data source

**Figure 1:**
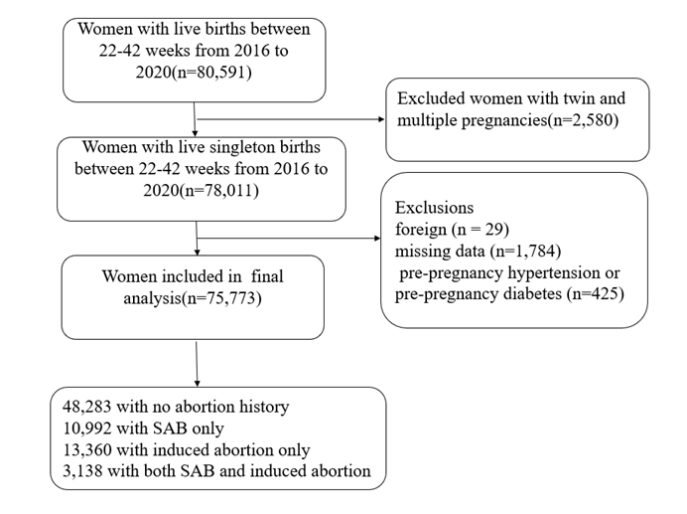
Flowchart of the process for pregnant women to join the group.

This retrospective cohort study was conducted at the Shanghai First Maternity and Infant Hospital, one of the largest prenatal care providers in Shanghai, China. A total of 80 591 pregnant women who gave birth to singleton live-born between 2016–20 were included in the study. Women with twins or multiple pregnancies, pre-existing hypertension or diabetes, or missing data on pregnancy history were excluded. Finally, 75 773 pregnant women were included in the analysis (**Figure 1**).

At the first prenatal visit, all women were interviewed by a trained nurse or obstetrician. Women’s social demographic characteristics, reproductive history (including parity, gravidity, SAB, IA), individual and family history of chronic diseases, and method of getting pregnant were all recorded in the medical register system. Pregnant women with SAB history, IA history or both SAB and IA were categorised into the exposed group, and the remaining pregnant women were included in the unexposed group. The primary outcome was birthweight, which was defined as LBW (<2500 g) [16] and macrosomia (≥4000 g) [17]. LBW was subdivided into very low birth weight (VLBW) (<1500 g) and mild low birth weight (1500–2500 g). The secondary outcome was preterm birth, which referred to delivering before 37 weeks of gestation.

### Statistical analysis

Characteristics of the study population were presented as n (%) according to abortion history. The χ^2^ test and univariate logistic regression models were used to analyse the statistical significance of the probability of LBW, macrosomia, VLBW, and premature birth among different abortion types. Multivariate logistic regression models were used to estimate the association of abortion history with the risk of adverse outcomes while adjusting for potential confounders. Furthermore, we investigated the associations between the number of abortion histories (one, two, three, or ≥4 times) and the risk of neonatal outcomes.

All statistical analyses were performed using SAS, version 9.4 (SAS Institute, Inc., Cary, North Carolina, USA). R, version 4.2.3 (R Core Team, Vienna, Austria) packages ‘ggplot2’, ‘plyr’, ‘RColorBrewer’, and‘foreign’ were used to draw figures. A two-sided *P*-value <0.05 was considered statistically significant.

## RESULTS

There were 75 773 pregnant women included in the final analysis, with 63.72% without history of abortion, 14.51% with SAB history, 17.63% with IA history, and 4.14% with both types of abortion history. The demographic characteristics of pregnant women by type of abortion are shown in **Table 1**. Women who experienced previous abortion(s) were more likely to be multiparous, with age ≥35 years, pre-pregnancy body mass index (BMI)≥25kg/m2, and gave birth by caesarean section compared with those without a history of abortion(s) (*P* < 0.05).

**Table 1:** Basic characteristics of the study population by the history of abortion or not*

Characteristics		History of abortion (n = 27 490)		SAB (n = 10 992)		IA (n = 13 360)	SAB and IA (n = 3138)		No abortion (n = 48 283)	
Age in years										
*≤24*	885 (3.22)		231 (2.10)		588(4.40)			66 (2.10)		2195 (4.55)
*25–29*	8411 (30.60)		3286 (29.89)		4427 (33.14)			698 (22.24)		22 284 (46.15)
*30–34*	11 866 (43.16)		5240 (47.68)		5256 (39.34)			1370 (43.66)		19 137 (39.64)
*≥35*	6328 (23.02)		2235 (20.33)		3089 (23.12)			1004 (31.99)		4667 (9.67)
Pre-pregnancy BMI in kg/m^2^										
*<18.5*	3592 (13.08)		1293 (11.76)		1960 (14.67)			339 (10.80)		7768 (16.09)
*18.5–25*	21 074 (76.66)		8472 (77.07)		10 173 (76.14)			2429 (77.41)		36 681 (75.97)
*≥25*	2824 (10.27)		1227 (11.16)		1227 (9.18)			370 (11.79)		3834 (7.94)
Parity										
*Nulliparous*	17 316 (62.99)		8551 (77.79)		7124 (53.32)			1641 (52.29)		39 811 (82.45)
*Multiparous*	10 174 (37.01)		2441 (22.21)		6236 (46.68)			1497 (47.71)		8472 (17.55)
Mode of delivery										
*Vaginal*	16 873 (61.38)		6737 (61.29)		8374 (62.68)			1762 (56.15)		34 185 (70.80)
*Caesarean section*	10 617 (38.62)		4255 (38.71)		4986 (37.32)			1376 (43.85)		14 098 (29.20)

BMI – body mass index, IA – induced abortion, SAB – spontaneous abortion

*Presented as n (%).

Compared with pregnant women who had no history of abortion, women who experienced SAB had an increased risk of delivering LBW and VLBW newborns and experienced preterm labour, with rates of 3.30 vs. 2.61%, 0.43 vs. 0.22%, and 0.76 vs. 0.45% (*P* < 0.05), respectively (**Table 2**). However, women who had experienced IA history had a lower probability of LBW and a higher occurrence of macrosomia (**Table 2**).

**Table 2:** Neonatal outcomes in the study population with different abortion classifications*

	History of abortion								
**Neonatal outcomes**	**SAB (n = 10 992)**	***P*-value**	**IA (n = 13 360)**	***P*-value**	**SAB and IA (n = 3138)**		***P*-value**	**No abortion (n = 48 283)**	
LBW	363 (3.30)	0.000	277 (2.07)	0.000	113 (3.60)	0.000			1258 (2.61)
Macrosomia	532 (4.84)	0.055	724 (5.42)	0.000	189 (6.02)	0.000			2134 (4.42)
VLBW	47 (0.43)	0.000	31 (0.23)	0.786	23 (0.73)	0.000			106 (0.22)
Premature birth	84 (0.76)	0.000	139 (1.04)	0.000	48 (1.53)	0.000			217 (0.45)

IA – induced abortion, LBW – low birth weight, SAB – spontaneous abortion, VLBW – very low birth weight

*Presented as n (%).

**Figure 2:**
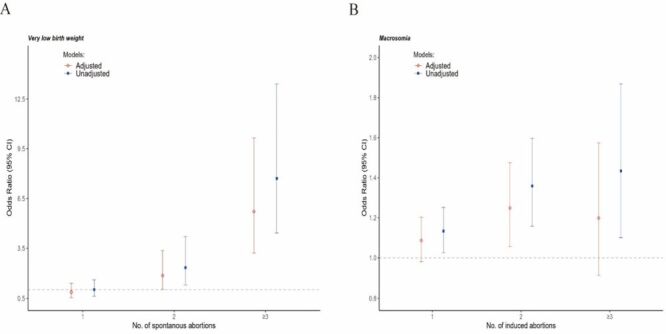
The correlation between the number of abortions. **Panel A.** Very low birth weight. **Panel B.** Macrosomia.

Adjusting for potential confounders, the odds of developing VLBW and preterm birth were odds ratio (OR) = 1.63 (95% confidence interval (CI) = 1.15–2.32) and OR = 1.38 (95% CI = 1.07–1.79), in women with a history of SAB compared with those without abortion history. The odds of LBW and macrosomia were OR = 0.76 (95% CI = 0.66–0.87) and OR = 1.16 (95% CI = 1.06–1.27) in women with a history of IA compared with those without an abortion history (**Table 3**). Furthermore, we observed that the likelihood of delivering a VLBW infant is associated with an increasing number of SABs. However, we did not find a correlation between preterm birth and the number of SABs. The risk of macrosomia increased with the increase of IA number. However, these risks attenuate insignificance after adjusting for confounding factors (**Figure 2**, **Table 4**, **Table 5**). The association between different types of abortions, the number of abortions, and more adverse outcomes are shown in Figure S1 in the **Online Supplementary Document**.

**Table 3:** Crude and adjusted OR (95% CI) for the associations between abortion classification and neonatal outcomes

Neonatal outcomes		History of abortion (n = 27 490), OR (95% CI)		SAB (n = 10 992), OR (95% CI)	IA (n = 13 360), OR (95% CI)		SAB and IA (n = 3138), OR (95% CI)		No abortion (n = 48 283), OR (95% CI)
LBW*			1.05 (0.96–1.15)	1.27 (1.13–1.43)		0.79 (0.69–0.90)	1.39 (1.14–1.69)	ref	
*Adjusted†*			0.95 (0.86–1.04)	1.11 (0.98–1.25)		0.76 (0.66–0.87)	1.19 (0.97–1.46)	ref	
Macrosomia*			1.20 (1.12–1.28)	1.10 (0.99–1.21)		1.23 (1.13–1.35)	1.38 (1.18–1.61)	ref	
*Adjusted†*	1.09 (1.02–1.17)			0.97 (0.88–1.07)		1.16 (1.06–1.27)	1.19 (1.01–1.40)	ref	
VLBW*	1.67 (1.27–2.20)			1.95 (1.38–2.75)		1.05 (0.70–1.57)	3.35 (2.13–5.27)	ref	
*Adjusted†*	1.36 (1.02–1.81)			1.63 (1.14–2.32)		0.94 (0.61–1.43)	2.48 (1.53–4.02)	ref	
Premature birth*	2.20 (1.84–2.63)			1.70 (1.32–2.19)		2.32 (1.88–2.88)	3.44(2.51–4.71)	ref	
*Adjusted†*	1.11 (0.93–1.34)			1.38 (1.06–1.79)		0.92 (0.74–1.15)	1.33 (0.96–1.84)	ref	

CI – confidence interval, IA – induced abortion, OR – odds ratio, ref – reference, SAB – spontaneous abortion, VLBW – very low birth weight

*Crude odds ratios and 95% confidence intervals.

†Adjusted according to the maternal age, pre-pregnancy body mass index, parity (nulliparous, multiparous), and mode of delivery (vaginal delivery, caesarean section).

**Table 4:** Crude and adjusted OR (95% CI) of the association between the number of SAB and neonatal outcomes

		Number of SABs, OR (95% CI)					
**Neonatal outcomes**	**1**			**2**		**≥3**	**No abortion (n = 48 283)**
LBW*	0.98 (0.85–1.13)		1.50 (1.20–1.88)		3.05 (2.33–3.99)		ref	
*Adjusted†*	0.90 (0.78–1.05)		1.22 (0.97–1.54)		2.25 (1.71–2.97)		ref	
Macrosomia*	1.06 (0.95–1.18)		1.13 (0.93–1.38)		1.21 (0.88–1.66)		ref	
*Adjusted†*	0.97 (0.86–1.08)		0.98 (0.80–1.21)		0.99 (0.72–1.37)		ref	
VLBW*	1.00 (0.63–1.59)		2.32 (1.28–4.20)		7.70 (4.42–13.40)		ref	
*Adjusted†*	0.87 (0.54–1.38)		1.84 (1.01–3.36)		5.71 (3.21–10.15)		ref	
Premature birth*	1.41 (1.05–1.89)		2.37 (1.56–3.61)		1.27 (0.52–3.09)		ref	
*Adjusted†*	1.10 (0.82–1.49)		2.18 (1.42–3.35)		1.25 (0.51–3.09)		ref	

CI – confidence interval, OR – odds ratio, ref – reference, SAB – spontaneous abortion, VLBW – very low birth weight

*Crude odds ratios and 95% confidence intervals.

†Adjusted according to the maternal age, pre-pregnancy body mass index, parity (nulliparous, multiparous), and mode of delivery (vaginal delivery, caesarean section).

**Table 5:** Crude and adjusted OR (95% CI) of the association between the number of IA and neonatal outcomes

		Number of IAs					
		**1**		**2**		**≥3**	**No abortion (n = 48 283)**
LBW*	0.72 (0.61–0.84)		0.99 (0.77–1.27)		1.13 (0.76–1.68)		ref	
*Adjusted†*	0.71 (0.61–0.84)		0.95 (0.74–1.22)		1.03 (0.69–1.54)		ref	
Macrosomia*	1.13 (1.02–1.25)		1.35 (1.15–1.59)		1.43 (1.10–1.86)		ref	
*Adjusted†*	1.08 (0.98–1.20)		1.24 (1.05–1.47)		1.19 (0.91–1.57)		ref	
VLBW	0.86 (0.53–1.40)		1.68 (0.88–3.21)		0.97 (0.24–3.93)		ref	
*Adjusted†*	0.81 (0.49–1.33)		1.46 (0.75–2.84)		0.71 (0.17–2.92)		ref	
Premature birth	1.43 (1.11–1.85)		2.65 (1.90–3.71)		5.06 (3.35–7.64)		ref	
*Adjusted†*	0.75 (0.57–0.97)		1.04 (0.74–1.46)		1.80 (1.18–2.74)		ref	

CI – confidence interval, IA – induced abortion, OR – odds ratio, ref – reference, SAB – spontaneous abortion, VLBW – very low birth weight

*Crude odds ratios and 95% confidence intervals.

†Adjusted according to the maternal age, pre-pregnancy body mass index, parity (nulliparous, multiparous), and mode of delivery (vaginal delivery, caesarean section).

## DISCUSSION

This retrospective cohort study of 75 773 singleton pregnant women in China. Our study revealed that women who experienced only SAB were at an elevated risk of VLBW and preterm birth. Conversely, women who experienced only IA were at a lower risk of LBW and had a higher risk of macrosomia. Furthermore, it was found that the likelihood of delivering a VLBW infant increased with an increasing number of SABs. This is one of the largest studies in China exploring the correlation between previous abortions and unfavourable neonatal outcomes.

Abortion can be divided into SAB and IA. Research has shown that the impact of varying types of abortion histories on the next pregnancies varies [11,18–20]. Several studies have demonstrated that women who have experienced previous SAB had a higher likelihood of experiencing preterm delivery in the next pregnancies [21–23]. This is in line with our findings that pregnant women with SAB history only had a higher probability of experiencing LBW, VLBW, and preterm delivery. A study conducted in Scotland has indicated that SAB history was associated with an increased risk of preterm delivery (OR = 1.26; 95% CI = 1.22–1.29), with the strongest effect size for extremely preterm birth (24–28 weeks of gestation) (OR = 1.73; 95% CI = 1.57–1.90). At the same time, the study also found that the more times SABs, the greater the risk of these unfavourable outcomes [21]. Although our study did not find a significant correlation between the times of SABs and preterm delivery, we did find that the risk of VLBW increased with the times of SABs for women with three or more SAB histories. Underlying mechanisms for miscarriage leading to preterm birth reported in studies included genetic factors and uterine or placental dysfunction [24]. Oliver-Williams et al. Suggested that the cervix and endometrium, mechanically damaged during SAB or IA procedures, might contribute to an elevated risk of preterm delivery in the next pregnancies [21,25,26].

Although we found a correlation between IA and the possibility of preterm birth, this association attenuate to insignificance after adjusting for potential confounders, which is in line with another study performed in Germany [27]. Other studies have also reported similar findings [28,29]. However, some studies have found an increased risk of preterm birth with an increasing number of IAs [20,30–32]. A study conducted in Finland found that a history of IA, especially multiple IAs, was linked to an elevated risk of extremely preterm delivery and LBW in the next pregnancies [29]. This finding is contrary to our study, as we found that women with IA history were at a lower risk of LBW and a higher risk of macrosomia. However, some studies have found no significant correlation between LBW and a history of IA [20]. Therefore, the limited data on LBW and the history of IA have produced conflicting results. Multi-centre and larger-scale studies are warranted to verify the relationship between LBW and history of IA.

Our study should be interpreted cautiously due to some limitations. First, as a retrospective-designed observational study, our study can only demonstrate an association between the history of abortion and neonatal outcomes instead of a causal relationship. Second, we did not conduct a subgroup analysis for preterm birth to examine the correlation between different classifications of preterm delivery and neonatal outcomes. Third, induced abortion can be categorised into two types – medical abortion and surgical abortion – which was not registered in the medical system in the hospital. We suggested further clarifying the classification of induced abortion history in future studies. Finally, because the data were all self-reported by women, the data on abortion, especially IA, may be underestimated.

In summary, previous studies have reported inconclusive and contradictory findings on the relationship between SAB history and IA history with the risk of preterm delivery [24,32,33]. The present study leveraged a large sample data set to explore the correlations between SAB and IA history and the adverse neonatal outcomes in subsequent pregnancies among Chinese pregnant women. Additionally, we recommend that future studies investigate the potential quantitative dependency between the abortion history and pregnancy outcomes.

## CONCLUSIONS

Our research indicates that pregnant women with a history of SAB are at an increased risk of delivering VLBW infants and experiencing preterm labour. The risk is positively associated with the number of SABs. Conversely, women with a history of IA are more likely to deliver macrosomic infants.

## Additional material


Online Supplementary Document
