# Supplementary material for: Association of different types of abortions with neonatal outcomes in subsequent pregnancy
Source: J Glob Health. 2024 Oct 18;14:04216. doi: 10.7189/jogh.14.04216 (PMC11487492; doi:10.7189/jogh.14.04216)
Supplement: Online Supplementary Document [file jogh-14-04216-s001.pdf]

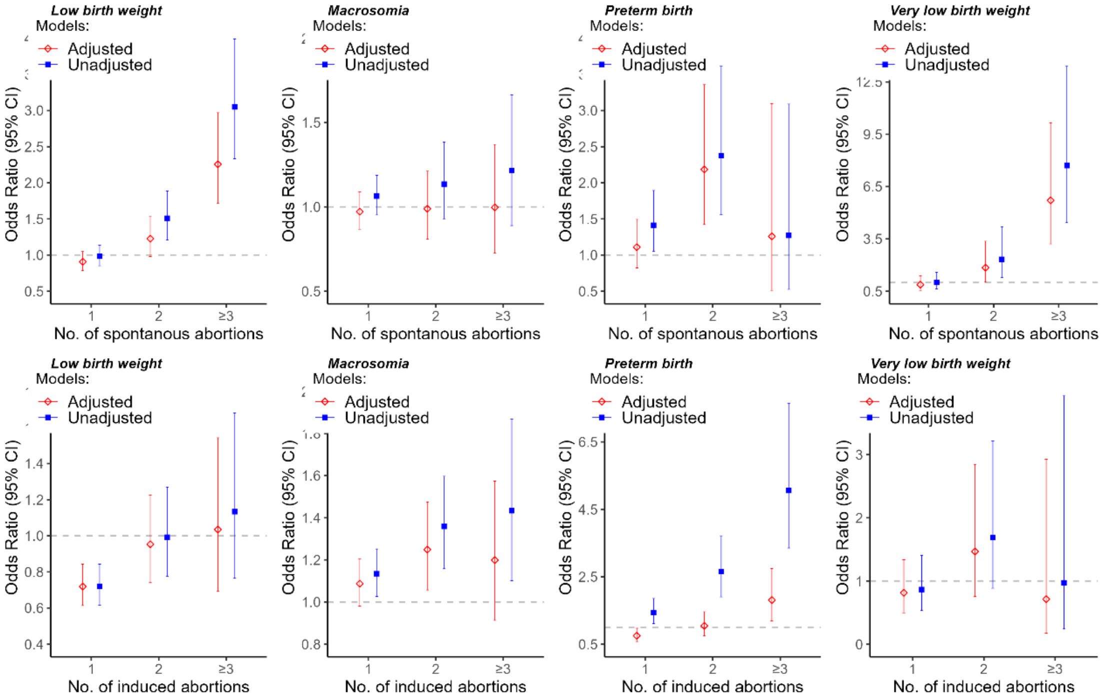

Supplementary Figure 1. The association between different types of abortions, the number of abortions, and adverse outcomes.
